# Une spondylodiscite tuberculeuse chez une transplantée rénale compliquée d’une mycose systémique

**DOI:** 10.11604/pamj.2014.19.22.2878

**Published:** 2014-09-09

**Authors:** Samira Bekaoui, Intissar Haddiya, Siham El Housni, Ryme El Harraqui, Hakima Rhou, Loubna Benamar, Fatima Eziatouni, Naima Ouzeddoun, Rabia Bayahia

**Affiliations:** 1 Service de néphrologie-dialyse-transplantation rénale, CHU Ibn Sina, Rabat, Maroc

**Keywords:** Transplantation rénale, spondylodiscite tuberculeuse, mycose systémique, Fluconazole, kidney transplantation, spinal tuberculosis, systemic mycosis, Fluconazole

## Abstract

En transplantation, les complications infectieuses sont fréquentes et de diagnostic souvent délicat. Elles peuvent coexister chez le transplanté rénal rendant leur diagnostic encore plus difficile. Le but de ce cas clinique est de discuter les difficultés diagnostiques et de surveillance de deux types de pathologies assez fréquentes chez le transplanté rénal, qui sont la tuberculose et la mycose, à travers l’observation clinique d’une patiente de 24 ans transplantée rénale qui présente une spondylodiscite tuberculeuse et qui développe secondairement une septicémie à Candida non albicans à point de départ urinaire dont le seul point d’appel est la fièvre post opératoire.

## Introduction

La fréquence de la tuberculose chez le transplanté rénal est 20 à 70% plus élevée par rapport à la population générale [1]. Elle se caractérise par la prédominance des formes extrapulmonaires et disséminées [2, 3]. La tuberculose vertébrale est une localisation inhabituelle chez le transplanté rénal. Les infections mycosiques invasives constituent une cause importante de morbi-mortalité chez la population transplantée. Nous rapportons l’observation d’une patiente transplantée rénale qui a présenté une spondylodiscite tuberculeuse ayant été compliquée d’une septicémie à Candida non albicans à point de départ urinaire.

## Patient et observation

Il s’agit d’une jeune patiente de 24 ans, transplantée en 2004 par un rein de donneur vivant apparenté. La fonction du greffon était normale avec une clairance de la créatinine à 114ml/min/(MDRD). Le traitement immunosuppresseur comprenait la Prédnisone( 5mg/j), la Ciclosporine (50mg x 2/j, la ciclosporinémie résiduelle: 60 - 80ng/ml) et le Mycophenolate mofétil (500mg x 3/j).

**Figure 1:**
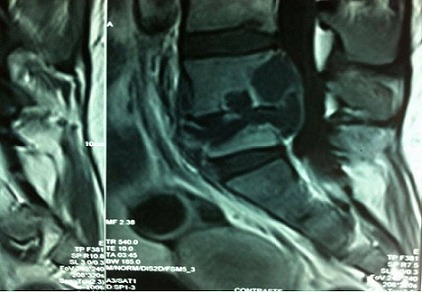
IRM mettant en évidence une spondylodiscite infectieuse au niveau L4-L5 et un abcès des parties molles paravertébrales antérieures à hauteur de L4-L5

Le début de La symptomatologie remonte à mars 2011 par des sciatalgies gauches. L’examen clinique a trouvé une patiente consciente, en bon état général, normotendue et apyrétique. La palpation des apophyses épineuses L4 et L5 était douloureuse. Le bilan biologique a montréune protéine C réactive (CRP) à 30 mg/l sans hyperleucocytose à la numération formule sanguine. L’intradermoréaction à la tuberculine (IDR) et le QuantiFERON-TB étaient négatifs. Par ailleurs, la fonction du greffon était conservée. La radiographie de la colonne lombaire était normale, l’imagerie par résonnance magnétique a mis en évidence une spondylodiscite infectieuse au niveau L4-L5 et un abcès des parties molles paravertébrales antérieures à hauteur de L4-L5 (Figure 1). La patiente a bénéficié d’une laminectomie L4-L5. Ella a été mise sous Ciprofloxacine et Métronidazole. Trois jours plus tard, la patiente a présenté une fièvre à 39-40C, malgré le traitement antibiotique. L’examen bactériologique de l’abcès paravertébra let la biopsie osseuse ont secondairement posé le diagnostic d’une spondylodiscite tuberculeuse. Le traitement antibacillaire était instauré, basé sur une quadrithérapie associant L’Isoniazide (5mg/Kg/j, 225mg/j), la Rifampicine(10mg/Kg/j, 450mg/J), l’Ethambutol (18.3mg/Kg/j, 825mg/j) et le Pyrazinamide (26.6mg/Kg/j, 1200mg/j). La durée prévue du traitement était de 12 mois.

L’évolution était marquée par la persistance de la fièvre malgré l’instauration traitement antituberculeux avec altération de l’état général en l’absence de stigmates d’infection patente. Au bilan biologique, la CRP était positive à 60mg /l puis progressivement à 150mg/l sans hyperleucocytose. La procalcitonine était positive à 10mg/l. Les prélèvements bactériologiques (l’étude cytobactériologique des urines, la ponction lombaire, les hémocultures sur milieux aérobique et anaérobique, la coproparasitologie des selles) étaient négatifs. La radiographie des poumons et l’échographie abdomino pelvienne étaient normales, le scanner thoraco-abdomino-pelvien a objectivé des images alvéolo-interstitielles diffuses non spécifiques.

Par ailleurs, la protéinurie était négative, la fonction du greffon était normale et la ciclosporinémie résiduelle était à 80ng/ml. Devant l’apparition de la fièvre sous Ciproxine et Métronidazole et sa persistance jusqu’ au 10^ème^ jour du traitement antibacillaire, une infection mycosique a été évoquée. L’étude mycologique des urines a isolé un Candida non albicans. L’identification de l’espèce du Candida et le fungigramme n’ont pas pu être réalisés. La patiente a été mise sous Fluconazole: 800mg/j. L’hémoculture sur milieu de Sabouraud a secondairement mis en évidence le même germe. Le diagnostic d’une septicémie à Candida non albicans à point de départ urinaire a été retenu. L’apyrexie a été obtenue 48 heures après le début du traitement antimycosique qui a été maintenu pendant 4 semaines. L’évolution a été marquée par l’amélioration clinique et biologique. La surveillance était complexe, elle portait sur quatre paramètres: 1) Le dosage sérique quotidien du taux de la Ciclosporinémie résiduelle. Sachant qu’elle a été abaissée par l’usage de la Rifampicine à un taux de 40ng/ml. Par conséquent, nous avons dû augmenter la dose de la Cyclosporine à 125mg x 2/j. Par la suite, l’introduction du Fluconazole a fait augmenter le taux de la Ciclosporinémie résiduelle à 200ng/ml ayant nécessité le retour à la dose habituelle de la Ciclosporine, soit 50mg x 2/j, qui par la suite a été ajustée après l’arrêt du Fluconazole. 2) La surveillance de la fonction du greffon qui était hebdomadaire pendant les deux premiers mois, puis mensuelle. Elle restait normale tout au long du traitement antituberculeux. 3) Le dosage sérique des antituberculeux (sauf l’Ethambutol) ayant été effectué au cours de la 1^ère^ semaine du traitement anti tuberculeux et qui a montré des taux sériques dans la fourchette cible. Ce dosage a été répété après l’introduction du Fluconazole, le taux sérique de la Rifampicine s’est abaissé à 4mg/l (cibles: 8-12mg/l), la dose a été augmentée de 150mg/j. 4) La surveillance du bilan hépatique qui était hebdomadaire pendant les 2 premiers mois, puis bimensuelle. Elle a montré une cytolyse hépatique au 4^ème^ mois qui a été respectée. L’examen ophtalmologique n’a pas révélé de signes retentissement de l’Ethambutol (névrite optique rétrobulbaire).

## Discussion

Nous rapportons l’observation d’une patiente transplantée rénale qui a présenté une spondylodiscite tuberculeuse ayant été compliquée d’une septicémie à Candida non albicans à point de départ urinaire.

Le patient transplanté rénal a un risque accru d’infection par les mycobactéries à cause du traitement immunosuppresseur qui altère la fonction de cytotoxicité des lymphocytes T, principal effecteur immunitaire contre ce type de germes [3]. La prévalence de la tuberculose chez le transplanté varie de 0.3 à 15% reflétant ainsi la variation de la l’incidence de la tuberculose chez la population générale dans chaque région [3-4]. Dans les pays d’endémie tuberculeuse, comme le Maroc, la fréquence de la tuberculose chez le transplanté rénal est 20 à 70% plus élevée par rapport à la population générale [1]. Quatre vingt quinze pour cent des cas surviennent pendant la 1^ère^ année suivant la greffe [5], la période ou l’immunosuppression et la corticothérapie sont maximales. IL peut s’agir d’une primo-infection ou d’une transmission par le greffon mais le plus souvent d’une réactivation à partir d’un foyer latent [6], favorisée par le traitement immunosuppresseur. Contrairement à la population générale, 30 à 50% des cas, sont des formes extrapulmonaires ou disséminées [2, 3]. Notre patiente a présenté une spondylodiscite tuberculeuse qui est une localisation inhabituellechez le transplanté rénal ayant survenu 8 ans après la greffe rénale. Il s’agit très probablement d’une réactivation à partir d’un foyer latent étant la localisation osseuse de l’infection tuberculeuse.

Le diagnostic peut être difficile à cause des signes cliniques qui sont atténués et atypiques. Ils peuvent se résumer à un amaigrissement, une asthénie et/ou une fébricule. L’intradermoréaction(IDR) à la tuberculine et les tests basés sur la production de l’IFN – γ peuvent être négatifs à cause de l’état de l’immunosuppression [3]. Le diagnostic n’est apporté parfois que par un examen invasif tel un tubage gastrique, un lavage bronchiolo-alvéolaire ou une biopsie. Notre cas clinique illustre ceci. La surveillance est délicate et complexe, elle porte sur l’efficacité et la toxicité des antituberculeux ainsi que sur leurs interactions médicamenteuses avec les immunosuppresseurs en particulier de la Rifampicine qui est un puissant inducteur enzymatique, elle interagit avec le cytochrome P450 et diminue la demie vie de nombreux médicaments à l’origine de la baisse de leur efficacité dont les inhibiteurs de la Calcineurine [7]. Le risque étant le rejet du greffon rénal. Chez notre patiente la dose de la Ciclosporine a été doublée. La Rifampicine diminue également l’aire sous la courbe du Mycophenolate mofétil et l’efficacité des corticoïdes [7]. L’association Rifampicine-antifongiques azolés diminue la biodisponibilité des deux medicaments et réduit leur efficacité [7]. La tuberculose du transplanté est grevée d’une lourde mortalité (20- 30%) et peut être à l’origine d’une perte du greffon [8]. L’évolution était bonne chez notre patiente. L’IDR à la tuberculine doit être réalisée chez tout candidat à la transplantation d’organe pour dépister une tuberculose latente, elle doit être refaite 15 jours plus tard si elle est négative [8]. Elle est considérée positive si l’induration est-supérieure ou égale à 5mm [4]. Le test de libération de l’interféron gamma peut être utile dans le dépistage de la tuberculose latente en cas de négativité de l’IDR à la tuberculine, cependant son rôle n’est pas établi chez la population des dialysés [4]. Les recommandations de EBPG (European Best Practice Guidelines) et la société américaine de transplantation recommandent de traiter la tuberculose latente par de la Rifampicine pendant 9 mois [9].

Le deuxième point soulevé par notre observation est l’infection fongique. Les mycoses invasives représentent une cause importante de morbi-mortalitéchez le transplanté [10]. Le Candidaspp et l’Aspergillusspp sont responsables de plus de 80% des cas de mycoses invasices chez le transplanté [11]. Leur diagnostic est difficile, il devrait être évoquée devant toute fièvre isolée résistante à une antibiothérapie à large spectre. Notre patiente a présenté une mycose systémique à Candida non albicans à point de départ urinaire favorisée probablement par la sonde vésicale qui aurait entrainé une dissémination sanguine du Candida. L’administration du Fluconazole chez notre patiente a compliqué la surveillance à cause de ses interactions médicamenteuses avec la Ciclosporine d’une part et avec la Rifampicine d’autre part. Le Fluconazole augmente la biodisponibilité de la Ciclosporine pouvant exposer le patient au risque de toxicité rénale. Fluconazole et Rifampicine l’un diminue l’efficacité de l’autre [8].

## Conclusion

En transplantation, les complications infectieuses sont fréquentes et de diagnostic souvent difficile à cause de leur présentation atypique. Elles peuvent coexister chez le transplanté rénal rendant le diagnostic encore plus difficile et le pronostic plus sombre. La tuberculose doit être évoquée devant tout signe atypique notamment dans une zone d’endémie. Toute fièvre isolée rebelle à une antibiothérapie à large spectre devrait faire rechercher une infection mycosique.
